# Supplementary material for: Light-Dependence of Formate (C1) and Acetate (C2) Transport and Oxidation in Poplar Trees
Source: Plants (Basel). 2022 Aug 9;11(16):2080. doi: 10.3390/plants11162080 (PMC9413118; doi:10.3390/plants11162080)
Supplement: Supplementary file 1 [file plants-11-02080-s001.zip › plants-1829496-supplementary.pdf]

Supplementary Material

Light-dependence of Formate (C_1) and Acetate (C_2) Transport and Oxidation in Poplar Trees

Kolby J. Jardine [1,*], Joseph Lei [1], Suman Som[1], Daisy Souza [2], Chaevien S. Clendinen [3], Hardeep Mehta [3], Pubudu Handakumbura [3], Markus Bill [1], Robert P. Young [3]

1*. Climate and Ecosystem Science Division, Lawrence Berkeley National Laboratory, Berkeley, CA, USA; kjjardine@lbl.gov

2. Forest Management Laboratory, National Institute for Amazon Research, Manaus, Brazil

3. Environmental Molecular Sciences Laboratory, Pacific Northwest National Laboratory, Richland, WA, USA

Supplementary Information Content

Supplementary Figure S1: Time series of headspace $\delta^{13}CO_2$ in a dynamic poplar branch enclosure during a DXSI experiment using a 10 mM solution of [^{13}C]formate.

Supplementary Figure S2: Time series of headspace $\delta^{13}CO_2$ in a dynamic poplar branch enclosure during a DXSI experiment using a 10 mM solution of [^{13}C]formate which is subsequently switched to a 50 mM solution of [^{13}C]formate.

Supplementary Figures

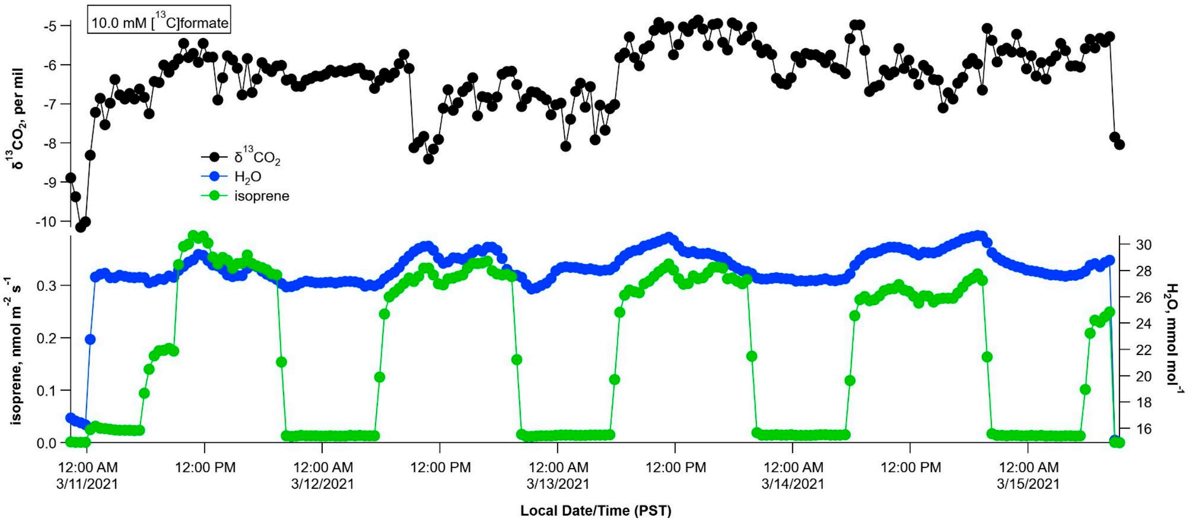

Figure S1: Time series of headspace H_2O water vapor concentrations, isoprene emissions, and headspace with $\delta^{13}CO_2$ in a dynamic poplar branch enclosure during a DXSI experiment using a 10 mM solution of [^{13}C]formate. The first and last part of the time series is an empty branch chamber.

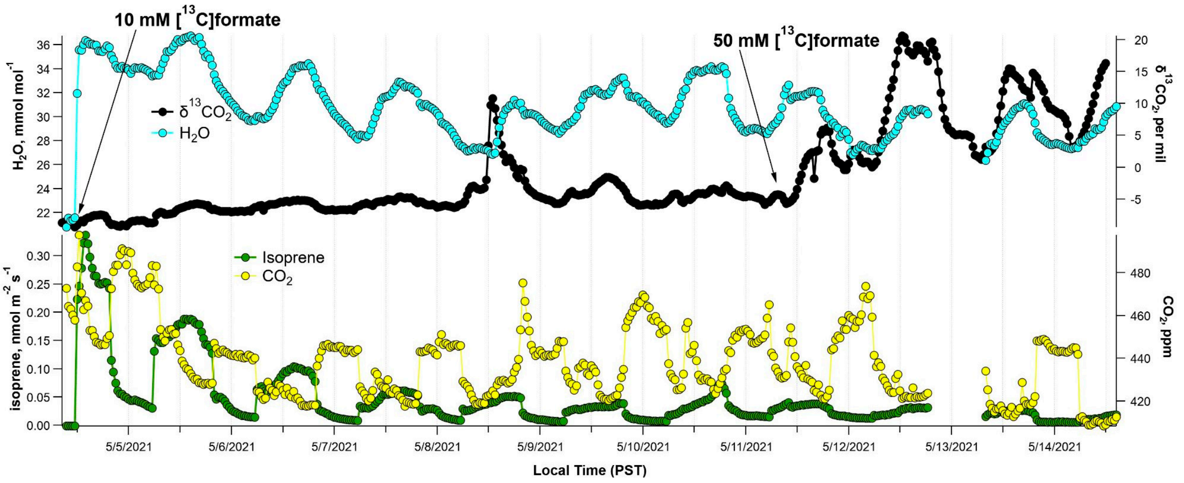

Figure S2: Time series of headspace H_2O water vapor concentrations, isoprene emissions, and headspace with $\delta^{13}CO_2$ in a dynamic poplar branch enclosure during a DXSI experiment using a 10 mM solution of [^{13}C]formate which is switched to a 50 mM solution of [^{13}C]formate (indicated by arrow). The first part of the time series is an empty branch chamber.
